# JAK1 inhibitor abrocitinib for the treatment of steroid-induced rosacea: case series

**DOI:** 10.3389/fmed.2023.1239869

**Published:** 2023-09-01

**Authors:** Bingyang Xu, Zining Xu, Shuhong Ye, Hong Sun, Bin Zhao, Na Wu, Jiawen Wu

**Affiliations:** ^1^Department of Dermatology, The Second Affiliated Hospital of Xi’an Jiaotong University, Xi’an, China; ^2^Department of Neurology, The Second Affiliated Hospital of Xi’an Jiaotong University, Xi’an, China; ^3^Department of Nursing, Xi'an Jiaotong University Medical School, Xi’an, China

**Keywords:** steroid-induced rosacea, JAK1 inhibitor, abrocitinib, case series, dermatology treatment

## Abstract

**Background:**

Steroid-induced rosacea is a severe withdrawal reaction which can occur after the frequent and excessive topical use of steroids on the face. The Janus kinase (JAK)-signal transducers and activators of transcription signaling pathway is involved in many biological processes and may play a role in the development of steroid-induced rosacea.

**Objective:**

To observe the efficacy and safety of the JAK1 inhibitor abrocitinib in the treatment of steroid-induced rosacea.

**Methods:**

Four Chinese female patients were treated with orally-administered abrocitinib, a selective JAK1 inhibitor with a good safety profile, for steroid-induced rosacea.

**Results:**

Abrocitinib treatment resulted in improved skin condition and lowered Dermatology Life Quality Index scores in each of the four patients. No discomfort was reported and no adverse effects were observed.

**Conclusion:**

The JAK1 inhibitor abrocitinib is a promising potential treatment for steroid-induced rosacea.

## Introduction

Steroid-induced rosacea is a severe withdrawal reaction which can occur after the frequent and excessive topical use of steroids on the face (1). It is characterized by rosacea-like dermatitis in the central face and perioral and periocular areas, manifesting as erythema, capillary dilation, scaling, papules, and pustules. It can be treated by discontinuing steroid use and with a variety of therapeutic agents, such as topical antibiotics, oral tetracycline antibiotics, and antihistamines; however, these traditional treatments are slow-acting and usually ineffective (2).

The Janus kinase (JAK)-signal transducers and activators of transcription (STAT) signaling pathway is stimulated by cytokines and involved in many important biological processes, such as cell proliferation, differentiation, apoptosis, and immune regulation (2). JAK1 inhibitors have been approved for the treatment of a variety of inflammatory skin conditions such as atopic dermatitis and psoriatic arthritis (3). The development of steroid-induced rosacea also involves the JAK–STAT signaling pathway. Previous studies have shown that steroids can induce Toll-like receptor (TLR) 2 gene expression; this may play an important role in steroid-induced acne and rosacea-like dermatitis (4). The selective JAK1 inhibitor class of drugs has a favorable safety profile, reducing the risk of inhibition of other JAK subtypes (5). In this paper, by reporting four cases of steroid-induced rosacea successfully treated with the JAK1 inhibitor abrocitinib, we show that abrocitinib is a promising potential therapeutic agent for the treatment of steroid-induced rosacea.

## Materials and methods

### Patients

All patients whose cases are presented here provided written informed consent to the publication of their cases.

Case 1: A 55-year-old woman presented with erythema with papules that had persisted for 2 years after the facial use of hormonal products. Repeated anti-inflammatory and antihistamine treatments had been ineffective, and persistent facial flushing and burning sensations in the skin caused distress. Upon initial presentation, the patient was treated with hydroxychloroquine, macrolide antibiotics, and a small dose of betamethasone intramuscularly. The patient reported relief from itching (Dermatology Life Quality Index [DLQI] score, 13), but erythema persisted.

Case 2: A 35-year-old woman presented with “rosacea” of 2 years’ duration, having previously self-administered hormone-based facial products. Initially, hydroxychloroquine, macrolide antibiotics, and low-dose betamethasone were administered intramuscularly. At the follow-up visit, the rosacea was in remission (DLQI score, 15), but papules and erythema were still present.

Case 3: A 35-year-old woman presented with persistent erythema and facial papules after topical hormone application for more than 1 year. She was experiencing increased facial erythema, dryness, and a burning sensation (DLQI score, 18).

Case 4: A 35-year-old female developed rosacea-like dermatitis on her face which persisted for more than 1 year after steroid discontinuation. Conventional treatments had only slight effects (DLQI score, 20).

### Physical examination

In this report, erythema, capillary dilatation, papules and pustules on an erythematous base, and enlarged follicular openings were seen on the facial skin of all four patients.

### Diagnosis

All four patients in this report underwent dermoscopy and octraspectral facial imagery testing. Dermoscopy revealed typical manifestations of rosacea, such as polygonal blood vessels on a red or purplish background, pustules centered on hair follicles, and perifollicular redness. The Octaspectral Facial Imaging System provides a holistic view of the distribution of blood vessels on the face and dynamically evaluates the severity of facial erythema in patients with rosacea as well as improvement before and after treatment. Combined with the patient’s symptoms and signs, and with reference to the results of auxiliary examinations, a diagnosis can be made. We also performed a Clinician’s Erythema Assessment (CEA) score.

All four of these patients’ CEA scores were between 3 and 4.

### Treatment

In all cases the patients professed a strong desire for the rapid improvement of symptoms, and therefore after careful evaluations we prescribed oral abrocitinib (100 mg once daily). In cases 2, 3, and 4, topical azelaic acid gel and a skin barrier protector were also applied during this period.

## Results

**Figure 1:**
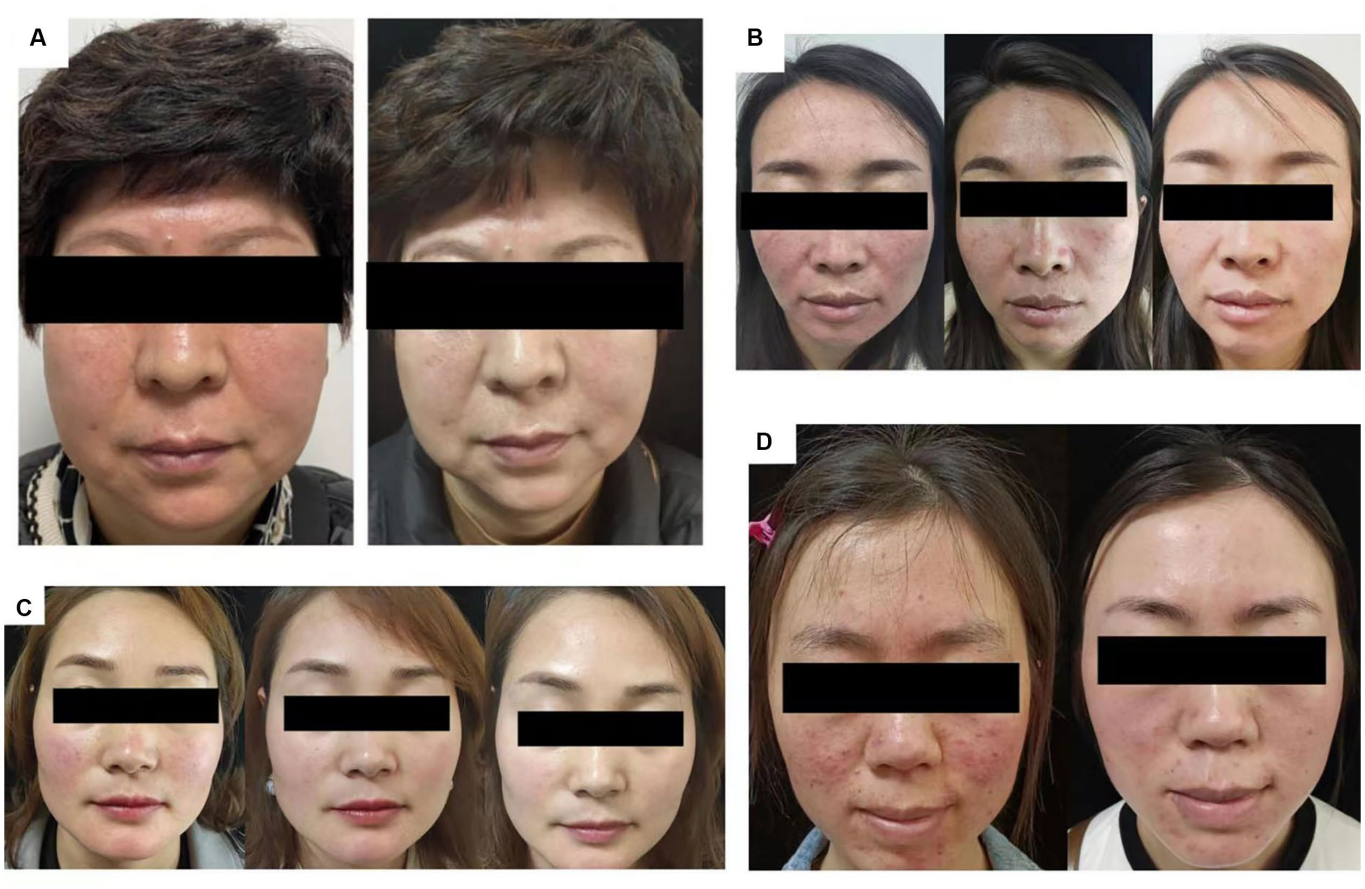
Skin condition of patients. **(A)** At 0 and 2 weeks of abrocitinib treatment. **(B)** Case 2, at 0, 2, and 4 weeks of abrocitinib treatment. **(C)** Case 3, at 0, 2, and 4 weeks of abrocitinib treatment. **(D)** Case 4, at 0 and 2 weeks of abrocitinib treatment.

After 2 weeks of treatment, the papules and facial erythema had improved significantly in all cases (Figure 1) and DLQI scores were 8, 7, 9, and 11 in cases 1, 2, 3 and 4, respectively. The patients did not report discomfort during treatment, and no adverse effects were observed.

**Figure 2:**
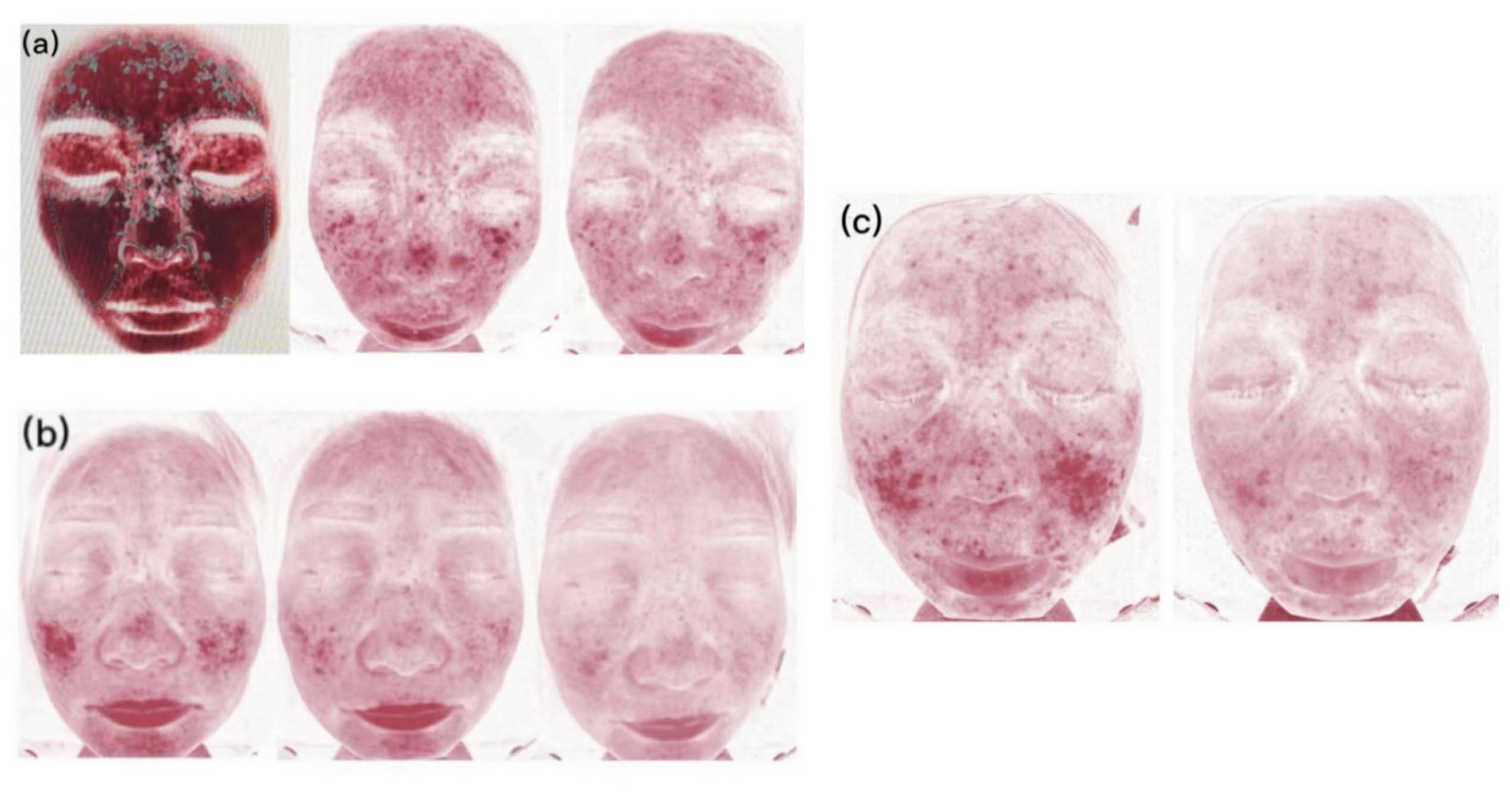
Skin vascularity of patients. **(A)** Case 2, at 0, 2, and 4 weeks of abrocitinib treatment. **(B)** Case 3, at 0, 2, and 4 weeks of abrocitinib treatment. **(C)** Case 4, at 0 and 2 weeks of abrocitinib treatment.

**Figure 3:**
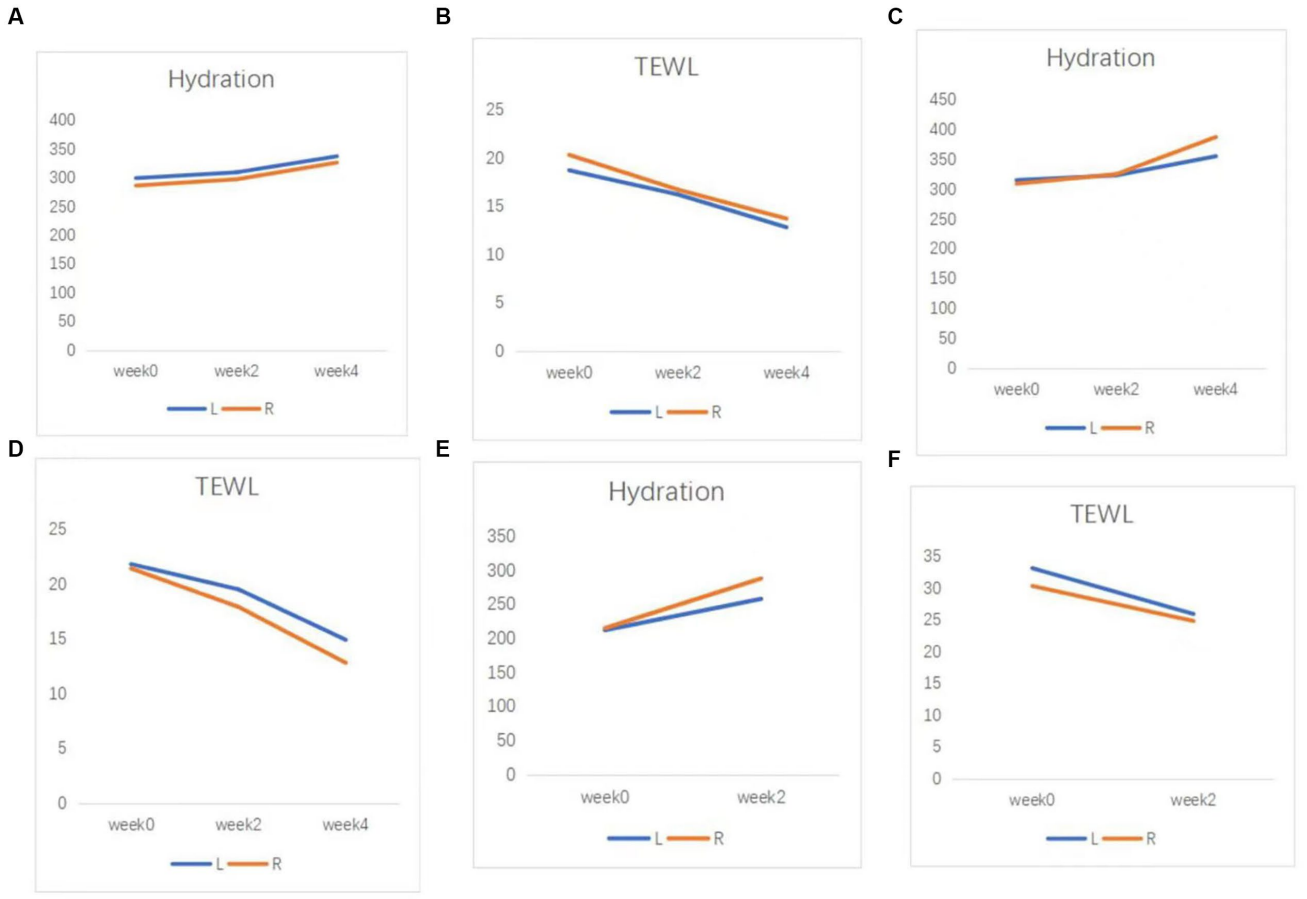
Skin barrier function of patients. **(A)** Hydration, and **(B)** TEWL results in case 2. **(C)** Hydration, and **(D)** TEWL results in case 3. **(E)** Hydration, and **(F)** TEWL results in case 4. TEWL, transepidermal water loss.

After treatment, CEA scores decreased to 0–2 in all four patients. The patients underwent assessment of vascular condition (Figure 2) and evaluation of skin hydration and transepidermal water loss as a measure of skin barrier function (Figure 3).

The patients in cases 2 and 3 have reached the 4-week follow-up with further improvements observed and DLQI scores of 5 and 6, respectively.

Follow-up of the patients in cases 2, 3, and 4 is ongoing. In case 2, the abrocitinib dose has been reduced to 100 mg orally every other day in an attempt to return to the conventional regimen.

Case 1 was lost to follow-up due to personal reasons. Case 2 showed continued improvement in facial erythema, a decrease in CEA score to 1, and improvement in facial vascularity, transepidermal water loss, and skin water content from the previous visit at week 6 follow-up after abrocitinib treatment. After a comprehensive assessment by the physician, the patient discontinued abrocitinib and was maintained on topical azelaic acid gel. At week 8 follow-up, the facial condition was stable and no adverse effects were complained of. In case 3, at the follow-up visit at week 6 after abrocitinib treatment, the CEA score had decreased to 2, and the relevant auxiliary examination indicators were improving, so the physician made a comprehensive assessment and continued the current regimen. At the follow-up visit at week 8, the CEA score decreased to 1, and the facial condition was stable. The patient discontinued abrocitinib and was maintained on topical azelaic acid gel. Case 4 continued to show improvement in facial erythema at week 4 and week 6 follow-up after abrocitinib treatment and did not complain of adverse effects, and is still adhering to the current regimen.

## Discussion

We report the cases of four patients treated with abrocitinib for steroid-induced rosacea with satisfactory results and no adverse effects.

The Eight Spectrum Facial Imager is a skin testing instrument that can reflect potential problems in the deeper layers of the skin by imaging with three light sources: white light, ultraviolet light, and polarized light. The degree of an inflammatory reaction of the facial skin and the area of lesions can be presented objectively and clearly, which can help provide clearer imaging of the changes in the lesions before and after t treatment (Figure 2). The moisture content of the skin is closely related to the structure of the stratum corneum, which is the site of the skin barrier function. It is not good to have too much or too little moisture in the stratum corneum. Transepidermal Water Loss represents the amount of water that evaporates from the surface of the skin; higher values indicate greater transepidermal water loss and poorer barrier function of the skin (Figure 3).

Steroid-induced rosacea is a severe withdrawal reaction that occurs after the frequent and excessive use of topical steroids on the face. It can be relieved by discontinuing steroids and with a variety of therapeutic agents such as topical antibiotics, oral tetracycline antibiotics, and antihistamines. However, due to the rebound phenomenon of hormones and the continued release of inflammatory factors, steroid-induced rosacea takes a long time to respond to these conventional treatments (6). Moreover, patients are often anxious, and we believe that this anxiety exacerbates lesions causing a vicious circle; therefore, more aggressive treatments are needed to induce rapid and effective anti-inflammatory effects to accelerate skin healing, improve patient mood, shorten treatment cycles, and stabilize the condition. From the results of the present study, we believe that oral abrocitinib may be a highly effective, safe, and stable treatment option for steroid-induced rosacea to stabilize the acute inflammatory phase more quickly.

The pathogenesis of steroid-induced rosacea remains unclear; however, some studies have suggested that it is related to the disruption of skin barrier function, vasodilatation disorders, and immune disorders. TLR2 is a classic intrinsic immune molecule that is involved in the pathogenesis of rosacea. Despite their immunosuppressive effects, steroids can increase the expression of the TLR2 gene in human keratinocytes, inducing various proinflammatory molecules to trigger skin inflammation (4). After steroid discontinuation, immunosuppression is lifted and proinflammatory factors are released in large amounts; this “inflammatory waterfall” effect may be the mechanism underlying the development of steroid-induced rosacea, making it difficult to treat. We believe that abrocitinib is effective in blocking the “inflammatory waterfall” effect, thus shortening the treatment cycle and facilitating the transition to conventional treatment.

**Figure 4:**
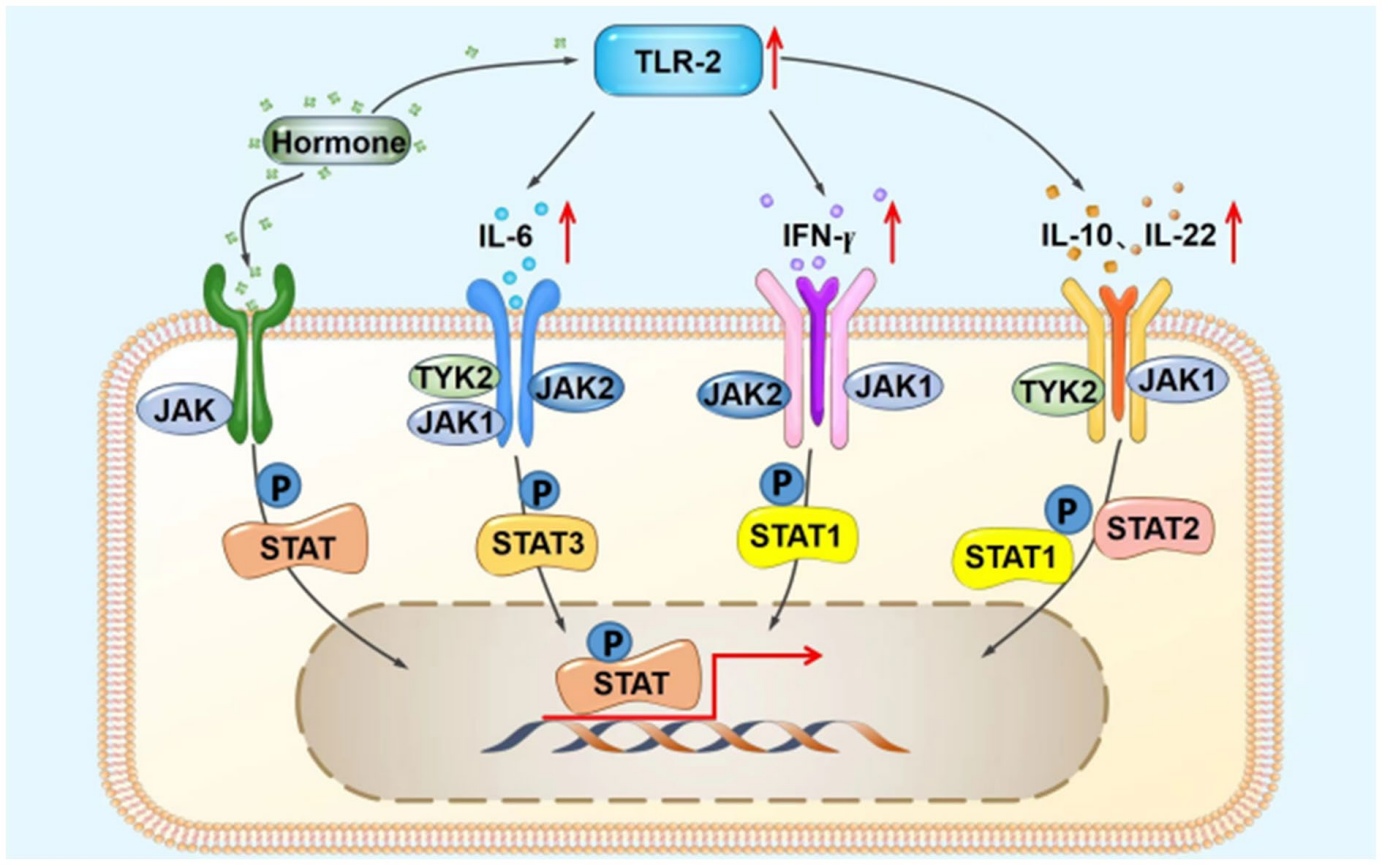
Possible schematic diagram of the interaction between the TLR2 and JAK/STAT signaling pathways. IFN-γ, interferon-γ; IL, interleukin; JAK, Janus kinase; STAT, signal transducers and activators of transcription; TLR2, Toll-like receptor 2; TYK2, tyrosine kinase 2; P, phosphorylation.

The JAK–STAT signaling pathway is a widely expressed intracellular signal transduction pathway stimulated by cytokines and involved in many important biological activities, such as cell proliferation and differentiation, apoptosis, and immune regulation. JAK is a common downstream element of several signaling pathways that mediate the response to a variety of inflammatory factors and cytokines such as interleukin (IL)-6, granulocyte-macrophage colony-stimulating factor, growth hormone, epidermal growth factor, IL-22, interferon (IFN)-γ, IL-31, IL-4, and IL-10 (7, 8). The JAK/STAT signaling pathway interacts with the TLR signaling pathway (9), as inflammatory mediators upregulated by TLR2, such as tumor necrosis factor-α, IL-6, and IL-8, induce the JAK/STAT signaling pathway (Figure 4). The TLR2 signaling pathway is an important mechanism for the development of steroid-induced rosacea, and IL-6, IL-10, IFN-γ, and other factors play an essential role (1, 4). Therefore, we speculate that JAK1 is important for the development of steroid-induced rosacea and that JAK1 inhibitors control the inflammation in steroid-induced rosacea through inhibition of the JAK/STAT signaling pathway.

Abrocitinib, a small-molecule inhibitor of JAK1, has been approved for the treatment of moderate-to-severe atopic dermatitis (10, 11). As mentioned in the reviewed paper, phase III clinical trials have demonstrated the effectiveness of abrocitinib in the treatment of moderate-to-severe atopic dermatitis (12). However, it is essential to acknowledge that real-life data are scarce. To further validate the efficacy and safety of abrocitinib, we recognize the necessity of real-world studies. For example, phase 3 trials have shown the efficacy and safety of il-23 inhibitors (e.g., guselkumab and risankizumab) in psoriasis treatment. Nevertheless, real-life data for these inhibitors remain limited. To address this, real-world, real-life studies conducted by Angelo Ruggiero, Matteo Megna, and others have successfully confirmed their efficacy and safety (13, 14). The cases in our report could provide novel and useful data on abrocitinib for real-world studies.

In addition, small-molecule JAK inhibitors have shown good efficacy in the treatment of various inflammatory skin diseases such as psoriasis and systemic lupus erythematosus (15). Additionally, research indicates that JAK inhibitors play a key role in the treatment of chronic inflammatory diseases such as psoriatic arthritis, hidradenitis suppurativa, and inflammatory bowel disease (16, 17). Previous studies have indicated that the JAK1/3 inhibitor tofacitinib can treat rosacea with satisfactory results. However, these two publications contained only one case of steroid-induced rosacea, and not all patients received tofacitinib monotherapy; some patients also received adjuvant therapy with conventional drugs (1, 18). All patients in the present study received abrocitinib monotherapy and presented with steroid-induced rosacea that had responded poorly to conventional therapy. In addition, abrocitinib, the selective JAK1 inhibitor used in this study, may have a higher affinity for inflammatory factors and has a good safety profile, with minimal inhibition of other JAK subtypes, reducing the risk of neutropenia and anemia. Based on the satisfactory outcomes of these four patients treated with abrocitinib, we suggest that the JAK1 pathway may play an important role in the pathogenesis of steroid-induced rosacea; however, the exact mechanism requires further investigation. We believe that oral abrocitinib is a promising treatment option for steroid-induced rosacea, as it is highly effective, safe, and stable.

## Conclusion

We report, to the best of our knowledge for the first time, the cases of four Chinese women with steroid-induced rosacea treated with the JAK1 inhibitor abrocitinib, with satisfactory results and no adverse effects. However, only a few cases are presented, the treatment time and follow-up time were short, and the specific mechanism of action is not clear; further studies on the efficacy and safety of abrocitinib in patients with steroid-induced rosacea are therefore needed.

## Data availability statement

The original contributions presented in the study are included in the article/supplementary material, further inquiries can be directed to the corresponding author.

## Ethics statement

Written informed consent was obtained from the individual(s) for the publication of any potentially identifiable images or data included in this article.

## Author contributions

JW and BX contributed to conception and design of the study. HS provided the design ideas. BX organized the data and wrote the first draft of the manuscript. ZX, SY, NW, and BZ were all involved in the process of data collection and analysis. All authors contributed to manuscript revision, read, and approved the submitted version.

## Funding

This study was supported by the Xi’an Jiaotong University Free Exploration Project (grant no. 2020YJ[ZYTS]309).

## Conflict of interest

The authors declare that the research was conducted in the absence of any commercial or financial relationships that could be construed as a potential conflict of interest.

## Publisher’s note

All claims expressed in this article are solely those of the authors and do not necessarily represent those of their affiliated organizations, or those of the publisher, the editors and the reviewers. Any product that may be evaluated in this article, or claim that may be made by its manufacturer, is not guaranteed or endorsed by the publisher.
